# Extracellular Vesicle Communication in the Central Nervous System: Mechanisms, Disease States, and Therapeutic Opportunities

**DOI:** 10.3390/bioengineering13101140

**Published:** 2026-09-30

**Authors:** Xiaoning Bi, Hanna Gootin, Wenyue Su, Michel Baudry

**Affiliations:** 1 College of Osteopathic Medicine of the Pacific, Western University of Health Sciences, Pomona, CA 91766, USA; hanna.gootin@westernu.edu (H.G.); wsu@westernu.edu (W.S.); 2 College of Dental Medicine, Western University of Health Sciences, Pomona, CA 91766, USA; mbaudry@westernu.edu

**Keywords:** extracellular vesicles, central nervous system, EV communication, state-dependent signaling, endolysosomal system, neurodevelopmental disorders, neurodegenerative diseases, EV biomarkers, EV therapeutics

## Abstract

Extracellular vesicles (EVs) are increasingly recognized as regulated mediators of intercellular communication in the central nervous system (CNS), transferring proteins, lipids, metabolites, and nucleic acids among neurons, glia, and other cells. EV communication comprises a complex integrated pathway that includes vesicle biogenesis, selective cargo sorting, secretion, extracellular transport, target-cell recognition, uptake, intracellular trafficking, functional cargo delivery, and recipient-cell responses. These processes are controlled by membrane trafficking machinery, cytoskeletal dynamics, lipid metabolism, calcium signaling, and the endosomal–lysosomal system and are further shaped by the physiological and pathophysiological states of donor and recipient cells. Genetic variation, proteotoxic stress, inflammation, metabolic and redox alterations, neuronal activity, and endolysosomal dysfunction can therefore affect multiple stages of the EV communication pathway in neurodevelopmental and neurodegenerative disorders. EV alterations reflect dynamic states rather than fixed disease signatures, with effects depending on cellular origin, EV subtype, anatomical context, developmental or disease state, and recipient-cell competence. This review examines CNS EV physiology and proposes a state-dependent framework for biomarkers and therapies, distinguishing EV detection from functional cargo delivery, cross-sectional associations from longitudinal progression, and cellular uptake from intracellular target engagement.

## 1. Introduction

Extracellular vesicles (EVs) are membrane-enclosed nanoparticles released by virtually all cell types, which mediate the transfer of proteins, lipids, metabolites, and nucleic acids between cells. Initially regarded as a mechanism for eliminating unwanted cellular components during reticulocyte maturation [1,2], EVs are now recognized as fundamental mediators of intercellular communication, which regulate development, tissue homeostasis, and repair across diverse organ systems [3,4,5,6]. In the central nervous system (CNS), EVs facilitate bidirectional communication among neurons, astrocytes, oligodendrocytes, microglia, vascular cells, and other types of cells, thereby contributing to synaptic plasticity, myelination, neuroimmune signaling, and circuit homeostasis [7,8,9].

EV research has expanded rapidly because of their potential as biomarkers and therapeutic delivery vehicles. Meanwhile, the research has also revealed substantial heterogeneity among EV populations. These advances prompted the development of the Minimal Information for Studies of Extracellular Vesicles (MISEV) guidelines. The guidelines recommend practices for EV nomenclature, isolation, characterization, and functional studies to improve rigor and reproducibility [10,11]. In addition to their translational applications, accumulating evidence indicates that EV biogenesis, cargo sorting, secretion, extracellular transport, cellular uptake, and intracellular trafficking are tightly regulated physiological processes integrated with membrane trafficking, endosomal–lysosomal function, cytoskeletal dynamics, and cellular signaling [12,13].

Scalable manufacturing is essential for translating EV-based therapies into clinical practice. Silva Couto et al. [14] adapted hTERT-immortalized human mesenchymal stromal cells to suspension culture, eliminating the need for microcarriers or other attachment matrices. These cells produced EVs in chemically defined medium, providing a platform for manufacturing process development. This approach could simplify scale-up, although clinical translation requires further validation of product consistency, safety, and potency within a GMP-compliant manufacturing process.

In the CNS, disruption of individual stages of the EV communication can alter neuronal and glial functions and contribute to neurodevelopmental, neurodegenerative, neuroinflammatory, and cerebrovascular disorders [7,15,16]. Increasing evidence indicates that EV composition and function are affected by the physiological and pathological states of both EV-producing and recipient cells [12,17,18]. Genetic variations, proteotoxic stress, inflammation, metabolic and redox alterations, neuronal activity, calcium signaling, and endolysosomal competence can modify EV biogenesis, cargo selection, secretion, trafficking, uptake, and recipient-cell responses [19,20,21]. These alterations may differ according to brain region, cellular origin, vesicle subtype, developmental timing, pathological burden, and recipient-cell competence [17,22]. We therefore propose a state-dependent framework in which EV signals are interpreted not as static and biologically uniform disease signatures but as components of a dynamic donor–EV–recipient communication pathway. This review builds on existing accounts of EV biogenesis, cargo, and disease associations by organizing these findings within a state-dependent donor–EV–recipient framework. The framework links donor-cell state to EV release and composition, and recipient-cell state to cargo processing and functional responses. Its distinctive contribution is to consider these factors jointly across development and disease progression. It therefore addresses the issue of when, where, and in which recipient cells an EV population produces a particular effect. This perspective also distinguishes benefits to donor cells from consequences for recipient cells, which may differ or oppose one another. From this perspective, we review the molecular mechanisms governing EV biogenesis, cargo sorting, secretion, extracellular transport, uptake, and intracellular processing and examine how EV state-dependent regulation influences CNS physiology, neurological disease, biomarker interpretation, and therapeutic responsiveness.

## 2. EV Classification and Biogenesis

EVs comprise heterogeneous populations generated through different membrane-remodeling pathways, including endosome-derived vesicles (exosomes; ~30–150 nm) and plasma membrane-derived microvesicles (~100–1000 nm). Apoptotic cells can additionally release larger membrane-enclosed particles (apoptotic bodies; ~500 nm–5 μm) during programmed cell death. By contrast, exomeres and supermeres represent recently described non-vesicular extracellular particles and should be distinguished from membrane-enclosed EVs. Because size, density, and individual protein markers cannot reliably establish vesicle origin, current consensus guidelines recommend the use of operational EV terminology unless biogenesis has been directly demonstrated [10,11,23,24,25]. Accordingly, this review focuses primarily on endosome-derived and plasma membrane-derived EVs, while distinguishing these populations from non-vesicular extracellular particles.

### 2.1. Exosome Biogenesis

Exosomes originate from the endosomal pathway. Endocytosis generates early endosomes that mature into late endosomes or multivesicular bodies (MVBs). During maturation, inward budding of the limiting membrane produces intraluminal vesicles (ILVs), which selectively incorporate proteins, lipids, metabolites, and nucleic acids. MVBs subsequently undergo one of two principal fates: fusion with lysosomes, resulting in degradation of ILVs and their cargo, or fusion with the plasma membrane, releasing ILVs as exosomes [1,2,6,26]. Rather than representing a single linear pathway, multiple MVB populations coexist within cells and differ in composition, intracellular localization, and secretory competence.

**Figure 1:**
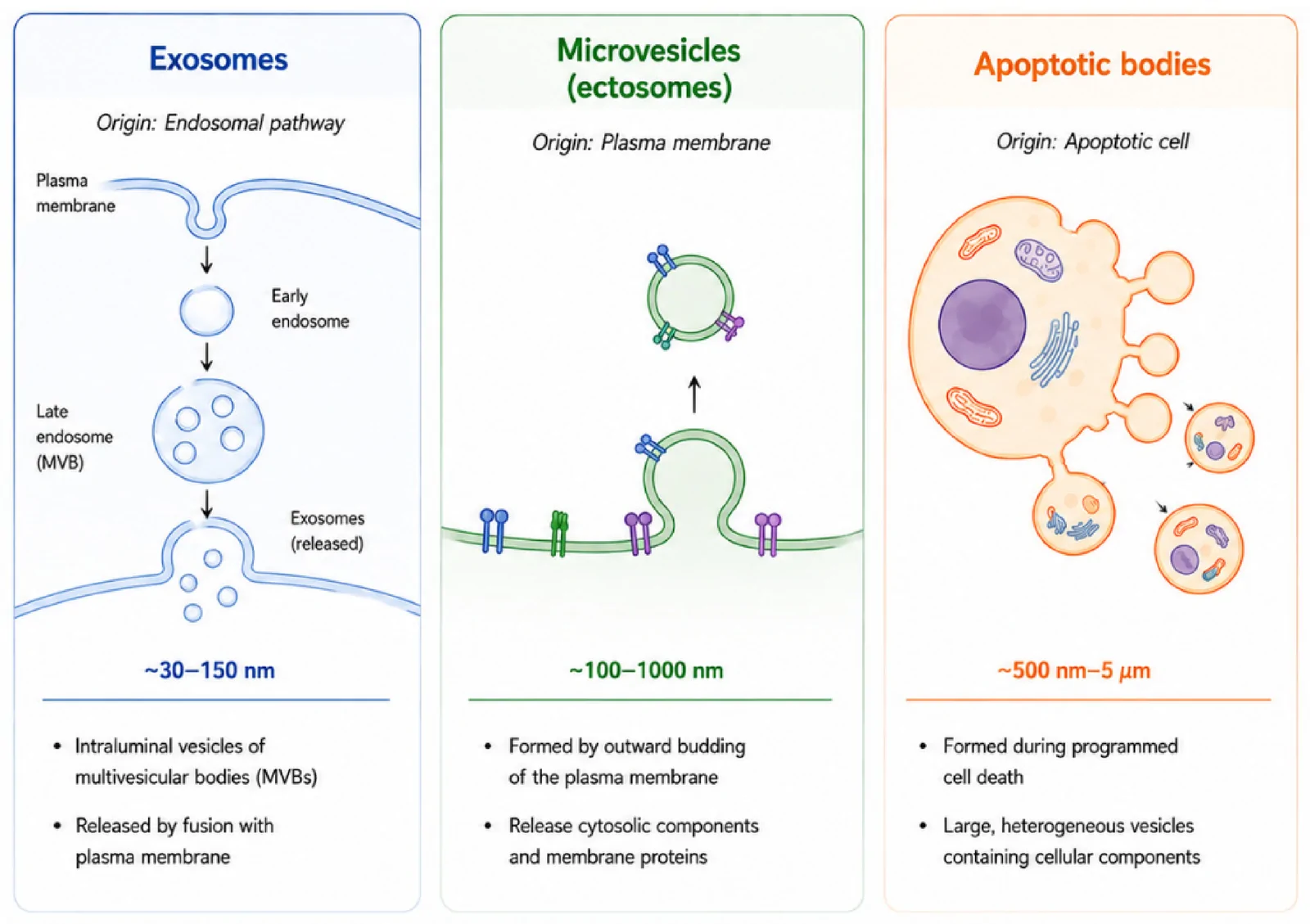
EV classification.

ILV formation is mediated by both ESCRT-dependent and ESCRT-independent mechanisms. In the canonical pathway, ESCRT-0, ESCRT-I, ESCRT-II, and ESCRT-III complexes sequentially recruit accessory proteins including HRS, TSG101, ALIX, VPS4, and CHMP proteins to coordinate cargo recognition, membrane invagination, and reverse-topology membrane scission [27,28,29]. However, not all ILVs require complete ESCRT machinery. Alternative pathways involving ceramide generated by neutral sphingomyelinase-2 (nSMase2), the syndecan–syntenin–ALIX complex, and tetraspanin-enriched membrane microdomains also contribute to ILV formation and cargo incorporation (Figure 1) [6,30,31,32]. These pathways cooperate rather than function independently, and their relative contributions vary among cell types and physiological conditions.

Cargo incorporation into ILVs is highly selective. Ubiquitination serves as an important sorting signal for many membrane proteins through interactions with ESCRT-associated proteins such as HRS and TSG101, whereas numerous proteins enter EVs through ubiquitin-independent mechanisms involving lipid microdomains, tetraspanins, adaptor proteins, RNA-binding proteins, and other post-translational modifications [12,16,28,33,34]. Consequently, EV composition reflects coordinated regulation by multiple sorting pathways rather than passive sampling of cellular constituents.

### 2.2. Microvesicle Biogenesis

Microvesicles (also referred to as ectosomes or shedding vesicles) arise directly from outward budding of the plasma membrane. Their formation requires coordinated remodeling of membrane phospholipids and the cortical cytoskeleton, including calcium-dependent activation of scramblases and calpain, actomyosin contraction mediated by Rho-family GTPases and ROCK, and membrane trafficking regulated by ARF6 and associated phospholipid signaling pathways [35,36,37,38,39,40,41]. These coordinated events drive membrane curvature, cargo clustering, and membrane scission to generate heterogeneous populations of plasma membrane-derived EVs. Although generally larger than exosomes, extensive overlap in size and molecular composition prevents reliable discrimination using physical properties or currently available protein markers [12,42]. Certain microvesicle populations also recruit ESCRT-associated proteins, including ARRDC1-mediated TSG101, indicating that ESCRT components contribute to selected plasma membrane budding events [28,43].

### 2.3. Lysosomal Regulation of Exosome Secretion

A central regulatory checkpoint in exosome biogenesis is the decision between lysosomal degradation and secretion. Following ILV formation, MVBs either fuse with lysosomes, leading to degradation of their cargo, or traffic to the plasma membrane where they fuse and release exosomes. Consequently, alterations in EV secretion frequently reflect changes in intracellular membrane trafficking rather than vesicle formation itself [12,13,26].

This degradative–secretory balance is coordinated by Rab GTPases, lysosomal calcium signaling, and nutrient-sensing pathways. Rab7 promotes late endosome maturation and lysosomal delivery, whereas Rab27a and Rab27b regulate transport, docking, and fusion of secretory MVBs with the plasma membrane [44,45,46]. In parallel, lysosomal Ca^2+^ release through the TRPML1 channel regulates lysosomal trafficking, membrane fusion, autophagy, and organelle positioning [47,48]. Nutrient sensing through the Ragulator–mTORC1–TFEB pathway further modulates lysosomal biogenesis and degradative capacity, thereby indirectly influencing EV secretion and cargo composition [47,49].

Recent evidence identifies LAMTOR1 as an upstream regulator of TRPML1-dependent lysosomal calcium signaling [50,51]. Beyond its established role in mTORC1 activation, LAMTOR1 regulates lysosomal positioning, membrane fusion, and cargo trafficking. These findings suggest that the LAMTOR1–TRPML1 axis may influence the balance between lysosomal degradation and EV secretion. However, its proposed role in coupling nutrient-sensitive lysosomal signaling to EV secretion remains to be directly established. We therefore propose that this pathway links intracellular membrane trafficking to extracellular communication. Angelman syndrome provides a disease context for investigating this possibility. UBE3A deficiency increases LAMTOR1 expression and disrupts lysosomal trafficking [20,52]. Forebrain synaptosomes from maternal UBE3A-deficient mice also exhibit reduced depolarization- or TRPML1-stimulated EV release compared with wild-type preparations [20]. These results demonstrate impaired stimulus-evoked EV release in this model. However, whether increased LAMTOR1 mediates the deficit remain to be determined.

Collectively, EV biogenesis should be viewed as an integrated membrane trafficking network rather than a series of isolated pathways. Endosomal sorting, plasma membrane remodeling, lipid metabolism, cytoskeletal dynamics, lysosomal trafficking, calcium signaling, and nutrient sensing collectively regulate vesicle formation, cargo selection, secretion, and extracellular communication [6,12,13]. This biogenesis-centered framework provides the conceptual basis for understanding the physiological functions of EVs in health and disease.

## 3. Cargo Sorting into EVs

The biological functions of EVs are determined not only by the number of vesicles released but also by their molecular composition. Proteins, lipids, nucleic acids, metabolites, and signaling molecules are selectively incorporated into EVs through coordinated membrane trafficking pathways rather than passive encapsulation [6,12,13,26]. Cargo selection begins during vesicle biogenesis and is governed by membrane identity, adaptor and scaffolding proteins, lipid microdomains, post-translational modifications, and RNA-binding proteins. The final cargo composition is further affected by endolysosomal trafficking, multivesicular endosome fate, and the physiological state of the donor cell, making EV cargo a dynamic reflection of cellular homeostasis rather than a random sample of intracellular contents [3,12,16].

### 3.1. Protein Cargo

Protein sorting is coordinated by ESCRT complexes, ALIX–syntenin interactions, tetraspanin-enriched membrane domains, and post-translational modifications including ubiquitination, phosphorylation, SUMOylation, glycosylation, and palmitoylation [6,28,30]. Recent quantitative proteomic studies demonstrate that EV protein composition differs substantially among EV subpopulations and reflects their intracellular origin rather than the simple enrichment of abundant cellular proteins [12,42,53]. In neurons, scaffold proteins, adhesion molecules, and synaptic organizers—including PDZ-domain proteins, Liprin family proteins, glypicans, and neurexin-associated proteins—are selectively enriched in EVs [53,54]. These protein classes have established roles in presynaptic and postsynaptic organization, supporting their contributions in synapse formation, plasticity, and circuit remodeling [55,56].

Beyond determining EV composition, selective protein export may regulate the intracellular abundance of synaptic proteins within donor neurons, as demonstrated in primary cortical neuron [57]. Similarly, selective EV export of tau has been shown to reduce tau accumulation within donor neurons while increasing pathogenic exposure in recipient cells [21]. Together, these findings demonstrate that EV cargo sorting and release regulate both extracellular protein transmission and intracellular proteostasis. EV proteomes may therefore provide information not only about intercellular signaling but also about neuronal activity, intracellular trafficking, proteostasis, and cellular stress within the donor cell.

### 3.2. RNA, Lipid, and DNA Cargo

EVs carry diverse RNA species, including mRNAs, miRNAs, lncRNAs, circRNAs, and other non-coding RNAs, which are selectively loaded by RNA-binding proteins such as hnRNPA2B1, YBX1, and SYNCRIP [5,34,58,59,60]. In the nervous system, EV-associated RNAs regulate neuronal differentiation, synaptic plasticity, neuroimmune signaling, and neuron–glia communication [7,15,61]. EV membranes are likewise enriched in cholesterol, sphingolipids, ceramide, phosphatidylserine, and glycosphingolipids, which contribute to membrane organization, vesicle stability, cargo clustering, and signaling functions [6,62,63]. In addition, EVs contain genomic and mitochondrial DNA, which appears to be incorporated preferentially under conditions of cellular stress, DNA damage, or impaired lysosomal and autophagic function and can activate innate immune pathways, including cGAS–STING signaling [3,53,64,65].

On the other hand, DNA detected in EV preparations (EV-associated DNA) may be intravesicular, surface-associated, or co-isolated with nonvesicular material. Its detection alone therefore does not establish encapsulation or functional delivery.

### 3.3. Exogenous Cargo Loading

Beyond endogenous cargo sorting, isolated EVs can be loaded with exogenous therapeutic cargo. Methods include passive incubation and membrane-permeabilizing approaches, such as electroporation, sonication, and extrusion [66,67]. Loading efficiency depends on cargo properties and the method used. These procedures may alter vesicle integrity or induce cargo aggregation.

Collectively, current evidence indicates that cargo incorporation is an actively regulated process, which integrates membrane trafficking, lipid organization, post-translational modifications, RNA-binding proteins, and cellular signaling [11]. Understanding how these mechanisms coordinate the selective loading of proteins, lipids, and nucleic acids will be essential for elucidating the roles of EV-mediated intercellular communication in physiology and disease.

## 4. EV Secretion

### 4.1. Exosome Secretion

Exosome secretion is a regulated exocytic process in which secretory MVBs are transported to, docked at, and fused with the plasma membrane, releasing intraluminal vesicles as exosomes. Rather than representing the final step of vesicle biogenesis, exosome secretion is a dynamic regulatory process that integrates intracellular trafficking, lysosomal function, calcium signaling, and extracellular stimuli [6,12,13,26].

Secretory MVB trafficking is coordinated by Rab GTPases, with Rab27A and Rab27B regulating vesicle docking and fusion, while Rab11 and Rab35 contribute to recycling endosome-dependent secretion in a cell- and cargo-specific manner [46,68,69]. Membrane fusion is mediated by SNARE proteins, including VAMP7, syntaxins, SNAP23, and their regulatory proteins, and is further modulated by intracellular Ca^2+^ and calcium sensors such as synaptotagmins [70,71]. In neurons, these mechanisms overlap extensively with the molecular machinery that regulates synaptic vesicle exocytosis, allowing neuronal activity to couple membrane trafficking with EV release.

Exosome secretion is highly responsive to physiological cues. Neuronal depolarization, synaptic activity, growth factor signaling, and metabolic status stimulate EV release, whereas inflammation, hypoxia, oxidative stress, and lysosomal dysfunction remodel both EV secretion and cargo composition [3,12,16,72,73]. Emerging evidence further indicates that lysosomal Ca^2+^ signaling mediated by TRPML1 and nutrient sensing through the Ragulator–mTORC1 pathway influence the balance between lysosomal degradation and exosome secretion, establishing secretion as an integral component of endolysosomal physiology rather than an isolated secretory pathway [47,50,51].

In excitable cells, electrical activity provides an important physiological link between cellular state and EV secretion. Membrane depolarization increases intracellular Ca^2+^ and engages trafficking and membrane-fusion machinery, thereby coupling electrical activity to EV release [72,73]. Although membrane potential is fundamental to neuronal physiology, direct evidence that resting membrane potential independently regulates EV composition or secretion remains limited. At present, activity-dependent and Ca^2+^-dependent regulation therefore provides a more firmly established mechanistic framework for understanding the relationship between neuronal excitability and EV secretion. 

### 4.2. Microvesicle Secretion

Microvesicles are released by outward budding and fission of the plasma membrane through coordinated remodeling of membrane lipids and the cortical cytoskeleton. Their secretion depends on localized changes in phospholipid asymmetry, membrane curvature, actomyosin contraction, and membrane scission, which together couple cellular activation to extracellular vesicle release [36,41].

These processes are regulated by intracellular Ca^2+^, phospholipid scramblases, calpain, ARF6, Rho-family GTPases, ROCK, and myosin light-chain kinase, which collectively coordinate membrane deformation and vesicle release [35,38,39,40]. Rather than functioning independently, these signaling pathways integrate mechanical forces, membrane organization, and cytoskeletal dynamics to regulate microvesicle secretion.

Like exosome secretion, microvesicle release is dynamically regulated by extracellular stimuli. Mechanical stress, inflammation, hypoxia, oxidative stress, and neuronal activity all enhance microvesicle shedding, enabling rapid adaptation of the extracellular signaling environment [13,41]. In the nervous system, activity-dependent microvesicle secretion contributes to neuron–glia communication, immune regulation, and synaptic remodeling, although the physiological functions and molecular specificity of plasma membrane-derived EVs remain less well understood than those of exosomes [7,15,61].

## 5. Extracellular Trafficking in the CNS

Following secretion, EVs travel through the brain extracellular space before interacting with recipient cells. Unlike soluble signaling molecules, EVs are membrane-bound nanoparticles whose movement is constrained by the unique architecture of the CNS, including the extracellular matrix (ECM), interstitial fluid flow, perivascular pathways, and cerebrospinal fluid (CSF). Together, these factors determine the spatial range, persistence, and specificity of EV-mediated communication within neural circuits [6,7,13,15].

### 5.1. Extracellular Transport in the Brain

Within the brain, EVs diffuse through the extracellular space but their mobility is regulated by tissue architecture, interstitial fluid dynamics, and neuronal activity. Consequently, EVs can function as local mediators of neuron–glia communication or undergo longer-range transport through CSF and perivascular pathways, enabling communication between anatomically distinct brain regions. EVs derived from neurons, astrocytes, oligodendrocytes, microglia, and choroid plexus are readily detected in CSF, highlighting its role as an important route for CNS-wide EV distribution [7,13,15].

### 5.2. Extracellular Matrix Regulates EV Mobility

The brain ECM is an active regulator of EV trafficking rather than a passive barrier. Heparan sulfate proteoglycans, hyaluronan, laminins, collagens, and other matrix components influence EV diffusion, retention, and interactions with recipient cells [74,75,76]. Dense extracellular-matrix structures such as perineuronal nets could potentially influence local EV mobility and accessibility; however, direct experimental evidence for such regulation remains limited.

### 5.3. Persistence and Clearance

The duration of EV signaling depends on the balance between extracellular transport, recipient-cell uptake, and clearance. Within the CNS, EVs are cleared through recipient-cell uptake, microglial phagocytosis, and drainage via cerebrospinal fluid and glymphatic pathways, which provide major routes for the removal of interstitial solutes from the brain [3,15,77,78]. The contribution of glymphatic pathways relative to other routes remains incompletely defined, particularly in humans. During neurodevelopment, aging, neuroinflammation, and neurodegeneration, alterations in ECM composition, glymphatic function, and glial activity can remodel EV distribution and persistence, thereby reshaping intercellular communication within neural circuits.

Collectively, extracellular trafficking is a dynamic determinant of EV signaling in the CNS. By regulating the movement, retention, and clearance of EVs within the brain microenvironment, extracellular transport establishes the spatial and temporal framework that governs EV-mediated communication in both physiological and pathological conditions.

## 6. Recognition, Uptake, and Intracellular Trafficking of EVs in the CNS

The physiological functions of EVs depend not only on their secretion and extracellular transport but also on their selective recognition by recipient cells, internalization, and intracellular cargo delivery. In the CNS, these processes determine how neurons, astrocytes, oligodendrocytes, microglia, and vascular cells exchange proteins, lipids, and nucleic acids to regulate synaptic plasticity, myelination, neuroimmune communication, and tissue homeostasis [6,7,13]. Rather than representing independent events, EV recognition, uptake, and intracellular trafficking form a continuous pathway that ultimately determines the specificity and functional outcomes of EV-mediated signaling.

### 6.1. Cellular Recognition and Uptake

EVs interact with recipient cells through the coordinated interactions between membrane proteins, lipids, glycans, and extracellular matrix components. Integrins, tetraspanins, immunoglobulin superfamily adhesion molecules, and heparan sulfate proteoglycans facilitate EV docking and help determine cell-type specificity [3,13,75]. In the brain, these interactions are further influenced by the extracellular matrix and perineuronal nets, which may locally regulate EV accessibility and signaling.

Following recognition, EVs enter recipient cells through multiple pathways, including clathrin-mediated endocytosis, caveolin-dependent uptake, macropinocytosis, phagocytosis, lipid raft-dependent internalization, and, in some cases, direct membrane fusion [6,13,79]. The predominant mechanism depends on both the EV and recipient cell types. Neurons can internalize small EVs through several endocytic mechanisms, whereas microglia also efficiently internalize EVs through phagocytic and macropinocytic pathways, consistent with their roles in immune surveillance and extracellular clearance [7,80]. Astrocytes and oligodendrocytes likewise both release and internalize EVs, supporting reciprocal neuron–glia communication and myelin homeostasis.

### 6.2. Intracellular Trafficking and Functional Cargo Delivery

Following internalization, EVs traffic through the endosomal system, where they may be recycled, degraded within lysosomes, or release their cargo into the cytoplasm [6,13]. Although many EVs are degraded, increasing evidence indicates that a subset delivers functional proteins, RNAs, lipids, and possibly DNA that modify gene expression, metabolism, and signaling in recipient cells. The mechanisms governing endosomal escape remain poorly understood and represent a major unanswered question in EV biology.

In the CNS, intracellular trafficking determines whether EVs regulate synaptic transmission, dendritic spine remodeling, axonal growth, myelination, or neuroimmune responses. Endolysosomal function is therefore a critical determinant of EV signaling. Lysosomal Ca^2+^ signaling mediated by TRPML1 regulates endosomal maturation, membrane fusion, and lysosomal dynamics [47,48]. We propose that the LAMTOR1–TRPML1 pathway not only regulates EV biogenesis and secretion but may also influence the intracellular fate of internalized EVs by controlling the balance between lysosomal degradation and functional cargo delivery. In neurological disorders characterized by endolysosomal dysfunction, including Angelman syndrome, altered intracellular trafficking may therefore impair both EV secretion and EV-mediated intercellular communication [20,50,51].

Importantly, detection of EVs within a tissue or recipient cell does not establish functional cargo delivery. Therapeutic studies must distinguish tissue distribution and cellular uptake from endosomal escape, intracellular target engagement, and a cargo-dependent biological response [81]. This distinction is particularly important in neurological disorders, where endolysosomal dysfunction may affect both EV production in donor cells and the intracellular fate of EVs in recipient cells. Demonstrating each step will be essential for determining whether an EV preparation reaches its intended target and produces its proposed therapeutic effect.

Despite substantial advances in identifying EV uptake pathways, the mechanisms governing cargo release, intracellular targeting, and functional integration remain poorly understood. Defining how recipient cells process internalized EVs will be essential for understanding CNS physiology and for developing EV-based therapeutics for neurological diseases.

## 7. Physiological Functions of EVs in the CNS

The CNS depends on highly coordinated communication between neurons, astrocytes, oligodendrocytes, microglia, endothelial cells, and other components of the neurovascular unit. In addition to classical neurotransmission and soluble signaling molecules, EVs provide a mechanism for transferring proteins, lipids, metabolites, mRNAs, microRNAs, and other regulatory molecules between neural cells, thereby integrating neuronal activity with glial support and immune surveillance [7,82,83,84,85].

During brain development, EVs regulate neural stem cell proliferation, neuronal differentiation, axon guidance, synaptogenesis, and circuit refinement through coordinated signaling between neural and glial cells. As neural circuits mature, EVs contribute to activity-dependent synaptic remodeling, dendritic spine maturation, neurotransmitter receptor trafficking, and homeostatic synaptic plasticity, thereby influencing learning, memory, and adaptive behavior [7,16,20,86]. Astrocyte-derived EVs provide metabolic substrates and neuroprotective molecules that support neuronal survival and synaptic function, whereas oligodendrocyte-derived EVs promote axonal integrity and myelin maintenance. Conversely, microglial EVs coordinate immune surveillance, synaptic pruning, and inflammatory signaling, linking neuronal activity with innate immune responses [83,84,85].

Emerging evidence further indicates that EVs participate in communication within the neurovascular unit. Endothelial cell-derived EVs influence blood–brain barrier (BBB) integrity, vascular remodeling, and neurovascular coupling, while brain-derived EVs can traverse the BBB and mediate bidirectional communication between the CNS and peripheral organs under both physiological and pathological conditions [87,88,89]. These findings suggest that EVs integrate local neural circuit function with systemic physiological regulation. It is noteworthy that the efficiency of EV BBB crossing depends on EV source, administration route, and barrier integrity. Brain-associated signals alone do not establish delivery of intact EVs or functional cargo to brain parenchymal cells.

EV secretion may support homeostasis within donor neurons rather than functioning solely as a pathway for intercellular cargo delivery. By regulating the intracellular abundance of synaptic and aggregation-prone proteins, EV export can contribute to neuronal protein homeostasis under both physiological and stressful conditions [21,57,90]. This function creates a potential tradeoff in which EV release reduces protein accumulation within donor cells while exposing recipient cells and connected neural circuits to the exported material. The biological outcomes therefore depend on the states of both the producing and recipient cells.

Collectively, EVs have emerged as essential regulators of CNS homeostasis by coordinating neuron–neuron, neuron–glia, glia–glia, and neurovascular communication. Through the dynamic exchange of bioactive cargo, EVs influence virtually every level of brain physiology, from neural development and synaptic plasticity to myelin maintenance, metabolic support, immune regulation, and brain–periphery communication. Disruption of these functions may therefore impair communication between neural cells while also altering the ability of donor cells to manage intracellular stress, providing a shared mechanism across neurological and neurodevelopmental disorders. These physiological functions are dynamic and can be modified by developmental stage, aging, metabolic state, and inflammation, which alter EV secretion, cargo composition, and recipient-cell responses. Thus, the functional consequences of EV communication depend not only on vesicle cargo but also on the physiological states of both donor and recipient cells.

## 8. EVs in Neurological Diseases and Therapeutic Opportunities

**Figure 2:**
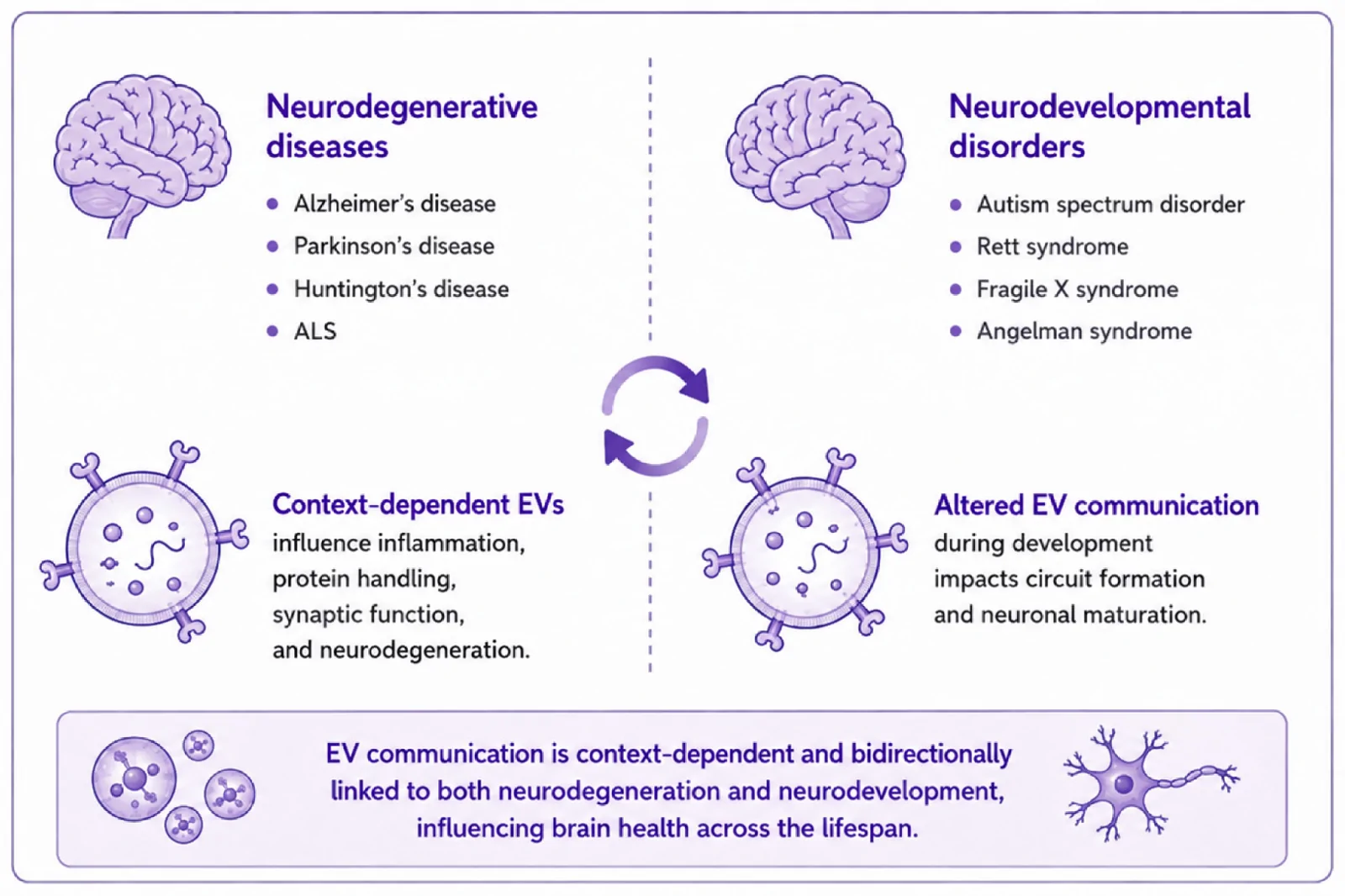
EV communication in neurodegenerative diseases and neurodevelopmental disorders is context dependent.

While EV-mediated communication is essential for maintaining neuronal and glial homeostasis, dysregulation of EV biogenesis, cargo sorting, secretion, extracellular trafficking, uptake, or intracellular cargo delivery can disrupt neural circuit function and contribute to disease initiation and progression. Importantly, EVs are not merely passive biomarkers of pathological processes but active participants that can either propagate disease or promote endogenous repair, depending on their cellular origin and molecular cargo [15,91]. This dual role has positioned EVs as both mechanistic mediators of CNS disorders and promising therapeutic platforms (Figure 2).

### 8.1. Neurodegenerative Diseases and Acute CNS Injury

Neurodegenerative diseases provide particularly informative settings for understanding how EV biology changes across disease states and how these changes may contribute to pathogenesis. Rather than representing a uniform increase or decrease in EV activity, disease-associated alterations can occur at multiple steps of the EV life cycle, including biogenesis, cargo sorting, secretion, trafficking, and recipient-cell signaling, and may vary with disease stage, brain region, cell type, and EV subtype. The following examples from AD, PD, HD, and ALS illustrate how EVs can simultaneously participate in proteostatic clearance, pathological protein propagation, and intercellular signaling while also providing molecular readouts of disease state and progression.

#### 8.1.1. Alzheimer’s Disease

Alzheimer’s disease (AD) is characterized by progressive amyloid-β and phosphorylated-tau pathology, accompanied by synaptic failure, neuronal loss, and cognitive decline. In cultured primary cortical neurons, Arc preferentially binds phosphorylated tau and cooperates with the membrane-remodeling protein IRSp53 to package seed-competent tau into neuronal EVs. Arc loss reduced EV-associated tau release, seeding, and neuronal transmission while increasing intracellular tau accumulation and early neuronal toxicity. Thus, Arc-dependent EV loading of tau illustrates a donor–recipient tradeoff in which EV-mediated export may reduce intracellular tau burden in donor neurons while promoting tau seeding and propagation in recipient cells [21].

EV alterations may also emerge before overt dementia. Neuronal-enriched circulating EVs from individuals with preclinical AD contained altered miRNA patterns, suggesting that EV cargo reflects biological changes occurring even before substantial cognitive impairment [92]. However, these cross-sectional differences do not establish that the same miRNA signals change continuously during disease progression. Moreover, EV populations isolated from AD brain tissue differed in both molecular composition and their ability to promote tau aggregation, demonstrating that total EV abundance can even conceal functionally distinct vesicle subtypes [93]. AD-associated EV signals must therefore be interpreted according to disease state, vesicle subtype, and biological activity rather than treated as a uniform marker of disease progression.

#### 8.1.2. Parkinson’s Disease

Parkinson’s disease (PD) involves progressive dopaminergic neuron loss and the accumulation of misfolded α-synuclein. In human dopaminergic neurons, elevated α-synuclein suppresses YKT6-dependent EV release and increases its own intracellular accumulation, showing that EV abnormalities may be consequences as well as mediators of proteotoxic stress [94]. Any attempt to restore secretion must therefore consider both donor-cell protein clearance and extracellular exposure of recipient cells.

Human biofluid studies further demonstrate why EV measurements should be interpreted in relation to clinical state. Oligomeric α-synuclein, an aggregation-prone form of the protein, was elevated in salivary EVs from individuals with PD and showed stronger diagnostic performance than total α-synuclein. Its levels were also associated with cognitive impairment and motor fluctuations. However, both EV measurements and clinical findings remained relatively stable over the one-year follow-up, supporting an association with clinical state rather than continuous progression tracking [95]. In contrast, repeated plasma measurements showed that within-person changes in EV-associated α-synuclein, tau, and amyloid-β were more closely related to changes in cognition and daily function than baseline levels alone [96]. Single-vesicle studies further suggest that the location of α-synuclein on or within an EV may provide information beyond its total concentration [97]. Together, these studies indicate that longitudinal change and vesicular context may be more informative than a single measurement of bulk EV cargo.

#### 8.1.3. Huntington’s Disease

Huntington’s disease (HD) is caused by an expanded CAG repeat in the HTT gene that produces aggregation-prone mutant huntingtin. Although the mutation is present throughout life, neuronal dysfunction develops gradually and progresses unevenly across brain regions, with the striatum showing particularly early vulnerability. This disease course provides an important model for determining whether EV changes vary with pathological severity, anatomical region, and vesicle subtype.

Analysis of HD brain tissue revealed that EV-associated alterations varied across brain regions and disease stages. Annexin A2, a membrane-associated protein linked to ectosome formation, was increased in cortical ectosomes at earlier neuropathological stages but approached control levels as pathology advanced. In contrast, striatal EV-associated output declined with increasing severity, accompanied by reduced levels of Alix, a protein involved in EV formation and cargo sorting [22]. These divergent patterns demonstrate that EV alterations in HD are region-, vesicle subtype-, and disease stage-dependent. Thus, the biological significance of an EV marker depends on its cellular and anatomical origin, the vesicle pathway from which it derives, and the pathological state of the tissue.

EV secretion may also participate in mutant huntingtin clearance from donor cells. IGF2 expression was reduced in peripheral blood from symptomatic compared with presymptomatic HD, suggesting a decline in this proteostatic pathway with disease progression [90]. In cellular models, IGF2 promoted EV-associated export of mutant huntingtin and reduced intracellular aggregate accumulation [90]. These findings suggest that EV secretion may serve as a proteostatic mechanism, which limits intracellular mutant huntingtin accumulation and that loss of such homeostatic support during disease progression could shift the balance toward intracellular retention and aggregation. However, current evidence is largely cross-sectional or derived from cellular models and does not establish a longitudinal EV trajectory or demonstrate that EV-associated mutant huntingtin contributes to long-range disease propagation in vivo.

#### 8.1.4. Amyotrophic Lateral Sclerosis

Amyotrophic lateral sclerosis (ALS) is a progressive motor-neuron disease involving multiple pathogenic mechanisms. Misfolded SOD1 and TDP-43 dysfunction can alter EV cargo through distinct pathways. Toxic trimeric SOD1 increased misfolded SOD1 on CD9-positive EVs, indicating that protein conformation can influence EV cargo sorting [98]. Whether this pathway contributes to pathogenic propagation in vivo remains unclear.

Human EV studies further suggest that distinct ALS mechanisms may produce distinguishable molecular signatures. Serum EVs from individuals with early-stage ALS showed altered protein and metabolite profiles, although their cross-sectional nature and unresolved cellular origins limit their value for monitoring progression [99]. A more mechanism-specific approach detected cryptic-exon-derived peptides in serum EVs from ALS patients associated with TDP-43 dysfunction [100]. Together, these findings support the potential of EV cargo to reveal distinct molecular mechanisms of ALS, but longitudinal and cell-specific validation remains necessary.

#### 8.1.5. Acute CNS INJURY and Epilepsy

Acute CNS disorders show how EV effects can change over time. After ischemic stroke or traumatic brain injury, EVs released by neural, vascular, and infiltrating immune cells regulate inflammation, blood–brain barrier integrity, angiogenesis, neurogenesis, axonal remodeling, and synaptic repair. Early responses may support containment and recovery, whereas persistent inflammatory EV signaling can worsen secondary injury. Seizure activity likewise changes EV release and cargo in epilepsy, with effects on inflammation, gliosis, and neuronal excitability [101,102,103,104,105,106]. These disorders reinforce the need to interpret EV signals according to the timing of injury and the balance between repair and ongoing tissue stress.

### 8.2. Neurodevelopmental Disorders

**Figure 3:**
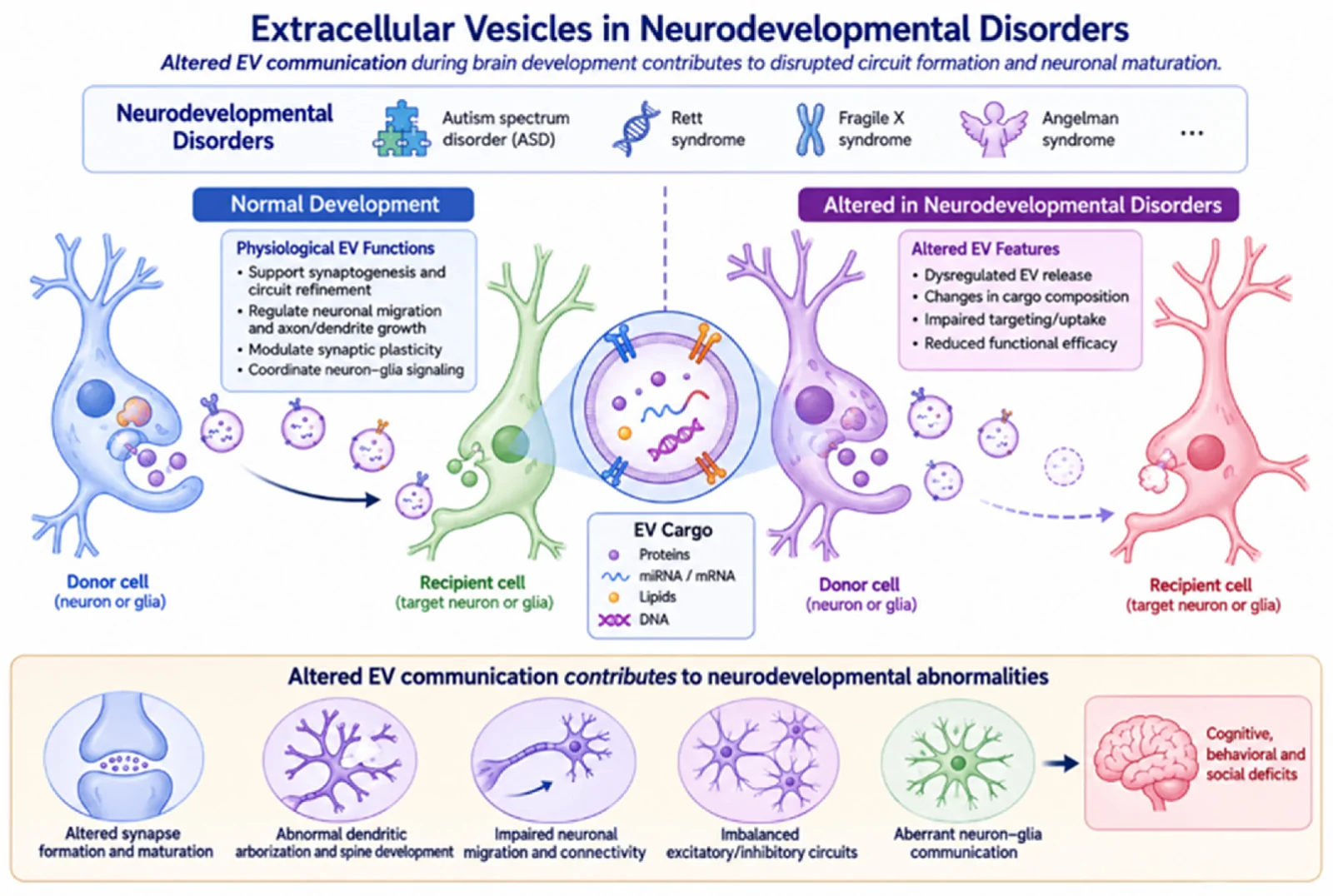
EVs in neurodevelopmental disorders.

Because EVs regulate neurogenesis, neuronal differentiation, axonal growth, synaptogenesis, synaptic pruning, and myelination, disruption of EV-mediated communication may profoundly affect brain development and neural circuit formation [8,107]. Unlike neurodegenerative diseases, neurodevelopmental disorders may alter EV signaling while neurons and glial cells are still differentiating, establishing synapses, and building functional circuits. Angelman syndrome, autism spectrum disorder, Rett syndrome, and Fragile X syndrome illustrate distinct ways that genetic mutations, altered developmental state, and inflammation can reshape EV release, cargo, uptake, and cellular effects (Figure 3).

#### 8.2.1. Angelman Syndrome

Angelman syndrome (AS) provides one of the clearest examples connecting a neurodevelopmental genetic state with defective EV communication. The disorder results from loss of neuronal UBE3A, an enzyme involved in protein turnover and intracellular trafficking [108,109,110]. Because the paternal copy of UBE3A is normally silenced in mature neurons [111], disruption of the maternal copy produces a substantial loss of neuronal UBE3A and contributes to abnormal synaptic development, intellectual disability, impaired speech, motor dysfunction, and seizures.

Maternal-Ube3a-deficient neuronal preparations exhibited lower activity- or TRPML1-stimulated EV release. TRPML1 was also required for efficient neuronal EV uptake. Treatment with wild-type neuronal EVs improved dendritic spine, synaptic, and cognitive abnormalities in an Angelman mouse model, indicating that EV dysfunction may contribute to the disease rather than merely accompany it [20]. These findings extend Angelman pathophysiology beyond intracellular signaling by showing that UBE3A deficiency may impair both the production of EV signals and their reception by neighboring neurons. However, the responsible therapeutic cargo, optimal developmental window, duration of benefit, tissue distribution, and relevance to humans remain unresolved.

#### 8.2.2. Autism Spectrum Disorder

Autism spectrum disorder (ASD) is a highly heterogeneous neurodevelopmental disorder involving diverse genetic risk factors and alterations in neuronal development, synaptic function, circuit connectivity, and immune regulation [112,113,114]. Consequently, a single EV cargo profile is unlikely to represent the entire spectrum. Instead, EV composition may reflect distinct developmental and inflammatory states among individuals with ASD.

Patient-derived forebrain organoids demonstrate that the developmental state of cortical tissue can alter the molecular messages carried by EVs. ASD-derived organoids showed increased progenitor growth and reduced neuronal development. Although their EVs did not differ substantially from control EVs in size or abundance, their RNA and protein cargo showed changes in pathways involved in gene regulation, protein synthesis, protein turnover, and synaptic function [115]. These findings demonstrate that apparently similar EV populations can carry different molecular information depending on the state of the developing tissue.

Peripheral EVs may also reflect and influence immune activity in ASD. Plasma EVs from patients with ASD contained altered inflammatory mediators and caused recipient lymphocytes to release more interferon-γ, a signal that promotes immune activation. Dexamethasone reduced ASD EV-induced IFN-γ secretion from recipient lymphocytes, demonstrating that the cellular response to disease-associated EVs can be pharmacologically modified [116]. Together, these studies suggest that EVs may reflect both altered cortical development and systemic immune activity. However, organoids do not reproduce complete brain maturation or systemic physiology, and plasma EVs cannot be assumed to originate from the CNS. These findings therefore support context-dependent EV alterations rather than a universal ASD signature.

#### 8.2.3. Rett Syndrome

Rett syndrome is a neurodevelopmental disorder caused in most cases by pathogenic variants in MECP2, which encodes methyl-CpG-binding protein 2 [117]. Following a period of apparently typical early development, affected children undergo developmental regression, typically with loss of acquired language, purposeful hand skills, and motor abilities [118]. This distinctive developmental course makes Rett syndrome particularly relevant for determining whether EV cargo changes dynamically across development rather than representing a static disease signature.

In isogenic forebrain organoids, EV-associated miRNA profiles changed as the neural tissue matured, and MECP2 deficiency produced developmental stage- and region-dependent alterations in the miR-302/367 cluster, which regulates pluripotency and differentiation [119]. These miRNAs were reduced in dorsal Rett organoids but increased in ventral organoids at later developmental stages, potentially reflecting altered differentiation and neuronal maturation. Although their causal contribution was not established, these findings demonstrate that Rett-associated EV cargo varies with developmental timing and regional identity.

Previous work also found that EVs from healthy neurons could partially improve neuronal maturation and synapse formation in MECP2-deficient human neurons [8,107]. Together, these studies suggest that Rett syndrome may involve both developmentally altered EV cargo and disruption of EV-mediated signals that support neuronal maturation, although the responsible cargo and its relevance in vivo remain unclear.

#### 8.2.4. Fragile X Syndrome

Fragile X syndrome results from loss of FMRP, an RNA-binding protein encoded by FMR1, which regulates protein synthesis at synapses [120,121]. Without FMRP, neurons develop abnormal synaptic protein expression and circuit activity, contributing to intellectual disability, sensory hypersensitivity, seizures, and autistic features [122,123]. Fragile X syndrome demonstrates how a neurodevelopmental mutation may impair EV secretion as a homeostatic function of the donor neuron.

Fmr1-knockout cortical neurons released fewer EVs and accumulated more CYFIP2, a regulator of synaptic structure. Stimulating EV secretion reduced intracellular CYFIP2, suggesting that EV export contributes to its homeostatic regulation [57]. FMRP deficiency may therefore disrupt synaptic function partly by impairing EV-mediated protein homeostasis, although whether CYFIP2-containing EVs influence recipient neurons, circuit development, or behavior in vivo remains unknown.

Related EV abnormalities have been reported in Down syndrome, where disrupted endosomal trafficking is associated with increased neuronal EV production and may alter the extracellular handling of neurotoxic proteins [8,107,124].

Collectively, these disorders illustrate distinct mechanisms by which neurodevelopmental state alters EV communication. AS links genetic mutations to impaired EV release and uptake; ASD to development- and inflammation-associated cargo remodeling; Rett syndrome to development- and region-dependent changes in EV cargo; and Fragile X syndrome to impaired EV secretion and donor-cell protein homeostasis. Thus, the biological significance of EV alterations depends on developmental timing, cellular origin, tissue context, and recipient-cell competence rather than diagnosis alone.

### 8.3. EV Biomarkers and Multimodal Interpretation

EV measurements may contribute to diagnosis, characterization of current disease state, prognosis, and treatment monitoring, but these functions should not be treated as interchangeable. A difference between clinical groups can establish an association with a defined disease state, whereas progression monitoring requires within-person EV changes that correspond with changes in clinical or biological function. EV measurements may therefore gain interpretive value when integrated with molecular biomarkers, imaging, cognition and motor function assessment, electrophysiology analysis, and other measures that independently define the relevant biological or clinical state.

Cell-of-origin validation is essential for circulating EVs described as “putative neuron-derived” or “neuronal-enriched.” Immunocapture alone does not establish neuronal provenance. Reports of predominantly soluble L1CAM contrast with single-vesicle evidence of L1CAM-positive EVs bearing neuronal markers [125,126,127]. Validation should confirm EV identity and marker colocalization, with controls for non-vesicular contaminants and nonspecific binding. Neuronal enrichment also does not establish CNS origin. Where provenance remains uncertain, preparations should be named by their isolation method. They may retain biomarker value, but disease associations alone cannot identify their cellular source.

Current studies illustrate these distinctions. Altered neuronal-enriched EV miRNAs in preclinical AD and serum EV multi-omic profiles in early ALS demonstrate that EV cargo can reflect a defined clinical or biological state, but neither cross-sectional finding establishes a progression trajectory [92,99]. In PD, salivary EV oligomeric α-synuclein and clinical measures both remained stable over one year, whereas a separate repeated-measures study found that within-person changes in plasma EV cargo were more closely related to changes in cognition and daily function than baseline levels alone [95,96]. HD brain tissue further showed that EV changes varied by neuropathological grade, brain region, and vesicle subtype, while TDP-43-related peptides in ALS EVs demonstrated the potential value of analyzing cargo linked to a defined molecular defect [22,100]. Together, these findings support multimodal interpretation as a framework for future validation, but they do not yet demonstrate that EV biomarkers can independently determine disease stage or predict disease progression. Table 1 summarizes biomarkers reported in major neurodegenerative diseases, their potential relevance, and interpretation limitations.

### 8.4. EV-Based Therapeutic Strategies

EVs have emerged as promising therapeutic platforms for CNS diseases because of their biocompatibility, relatively low immunogenicity, capacity to protect and transport diverse molecular cargo, and potential to interact with and cross biological barriers, including the blood–brain barrier (BBB) [128,129] (Table 2). EV preparations show limited immune activation under tested conditions. However, low immunogenicity is not an intrinsic property of all EVs and depends on their source, composition, purity, dose, and dosing schedule. EVs derived from mesenchymal stem cells, neural stem cells, neurons, and glia can carry trophic, anti-inflammatory, and regenerative signals, whereas engineered EVs can be loaded with therapeutic proteins, nucleic acids, or small molecules modified to enhance tissue or cell targeting [15,130,131,132].

Across preclinical neurodegenerative models, EV interventions target inflammation, oxidative stress, trophic signaling, mitochondrial dysfunction, and proteinopathies. However, differences in EV source, cargo, dose, administration route, disease model, and treatment timing prevent direct comparison across studies [133]. In three months old 5xFAD mice, intranasal administration of neural-stem-cell EVs improved measures related to oxidative stress, mitochondrial function, cellular clearance pathways, BDNF signaling, and neurogenesis [134]. These findings suggest that an early AD-associated environment retains several pathways responsive to EV treatment, although the absence of later-stage comparisons and identification of the active cargo precludes conclusions about stage-specific efficacy.

**Table 2:** Comparison of therapeutic EV strategies in CNS diseases.

Disease	EV source/Platform	Therapeutic Cargo	Administration Route	Model/Population	Target-Engagement Evidence	Reported Outcomes	Key Limitations	Reference
AD	Neural stem cell-derived EVs	Endogenous cargo; active components unresolved	Intranasal	Three-month-old 5xFAD mice	Changes in BDNF signaling and other cellular pathways; direct engagement by a defined cargo not established	Improvements in measures of oxidative stress, mitochondrial function, cellular clearance, and neurogenesis	No later-stage comparison; active cargo unresolved; stage-specific efficacy and human relevance uncertain	[134]
ALS	Liver-directed platform generating circulating RVG-tagged EVs	siRNA targeting mutant SOD1	Liver-directed platform delivery; EV production in vivo	Mutant SOD1 ALS mouse model	Neuronal delivery and reduction in the intended SOD1 target support functional siRNA activity	Improved motor, neuromuscular, inflammatory, and survival outcomes	Limited applicability beyond SOD1-associated ALS; safety, dose control, off-target effects, and long-term feasibility unresolved	[135]
AS	Healthy neuron-derived EVs	Endogenous cargo; active components unresolved	Intravenous	AS experimental model	Neuronal and behavioral rescue supports biological activity; a specific cargo–target mechanism remains unresolved	Improved neuronal and behavioral deficits	Active cargo, therapeutic window, durability, and human relevance unresolved	[20]
AD	Allogeneic adipose MSC-derived EVs	Endogenous cargo; active components unresolved	Intranasal	Patients with AD in a small exploratory clinical study	Direct CNS cargo delivery and molecular target engagement not established	No adverse events reported; preliminary cognitive findings	Small exploratory study; efficacy requires larger controlled trials; long-term safety and mechanism remain uncertain	[136]; NCT04388982

Abbreviations: AD, Alzheimer’s disease; ALS, amyotrophic lateral sclerosis; AS, Angelman syndrome; EV, extracellular vesicle; MSC, mesenchymal stromal cell; RVG, rabies virus glycoprotein; siRNA, small interfering RNA. Target-engagement evidence is distinguished from EV uptake, tissue accumulation, pathway changes, and phenotypic improvement. “Unresolved” indicates that the supplied discussion does not establish the mechanism.

Engineered EVs illustrate the additional requirements for targeted cargo delivery. In an ALS mouse model, a liver-directed platform generated circulating RVG-tagged EVs carrying an siRNA against mutant SOD1. These EVs reached spinal cord and cortical neurons, reduced levels of the intended molecular target, and improved motor, neuromuscular, inflammatory, and survival outcomes [135]. This study connects EV production and circulation with neuronal targeting, intracellular cargo activity, and functional improvement. Nevertheless, the SOD1 model represents only a subset of ALS, and the safety, dose control, off-target effects, and long-term feasibility of the platform require further evaluation.

Angelman syndrome provides a complementary strategy in which treatment attempts to restore physiological EV communication rather than deliver engineered cargo. Administration of EVs from healthy neurons improved neuronal and behavioral deficits in an AS mouse model, supporting the therapeutic potential of restoring EV-mediated signaling, although the active cargo, therapeutic window, durability, and human relevance remain unresolved [20].

EV-based therapies have entered clinical investigation for neurological diseases, including AD, PD, and stroke (Table 3). A small exploratory AD study reported no adverse events following intranasal administration of allogeneic adipose mesenchymal stromal cell (MSC)-derived EVs [136]. Preliminary cognitive findings require confirmation in larger controlled trials. Other registered studies are evaluating intranasal MSC-derived EVs and intravenous induced pluripotent stem cell-derived EVs. These efforts demonstrate early clinical translation, but therapeutic efficacy remains unestablished.

Therapeutic success ultimately will require more than EV accumulation in the target tissue. EVs must reach the intended cells, deliver functional cargo, engage the proposed intracellular target, and produce a cargo-dependent response within the relevant disease environment. Clinical translation will also require well-defined EV preparations, complementary characterization methods, control of co-isolated materials, justified dose metrics, stability and biodistribution studies, and reproducible manufacturing, and mechanism-linked potency assays [11,81]. Therapeutic equivalence should therefore consider not only EV source, cargo, and dose but also whether recipient tissues retain the cellular machinery required for uptake, intracellular processing, target engagement, and biological response.

## 9. Conclusions and Future Perspectives

EVs regulate diverse aspects of CNS physiology, including neural development, synaptic plasticity, neuron–glia communication, neurovascular function, immune surveillance, and tissue homeostasis. These functions depend on an integrated pathway encompassing EV biogenesis, cargo selection, secretion, extracellular transport, recipient-cell uptake, and functional cargo delivery. EVs should therefore be viewed as active components of the communication networks that coordinate brain function across cells, regions, and stages of life [6,7,15,16,83,84,85].

**Figure 4:**
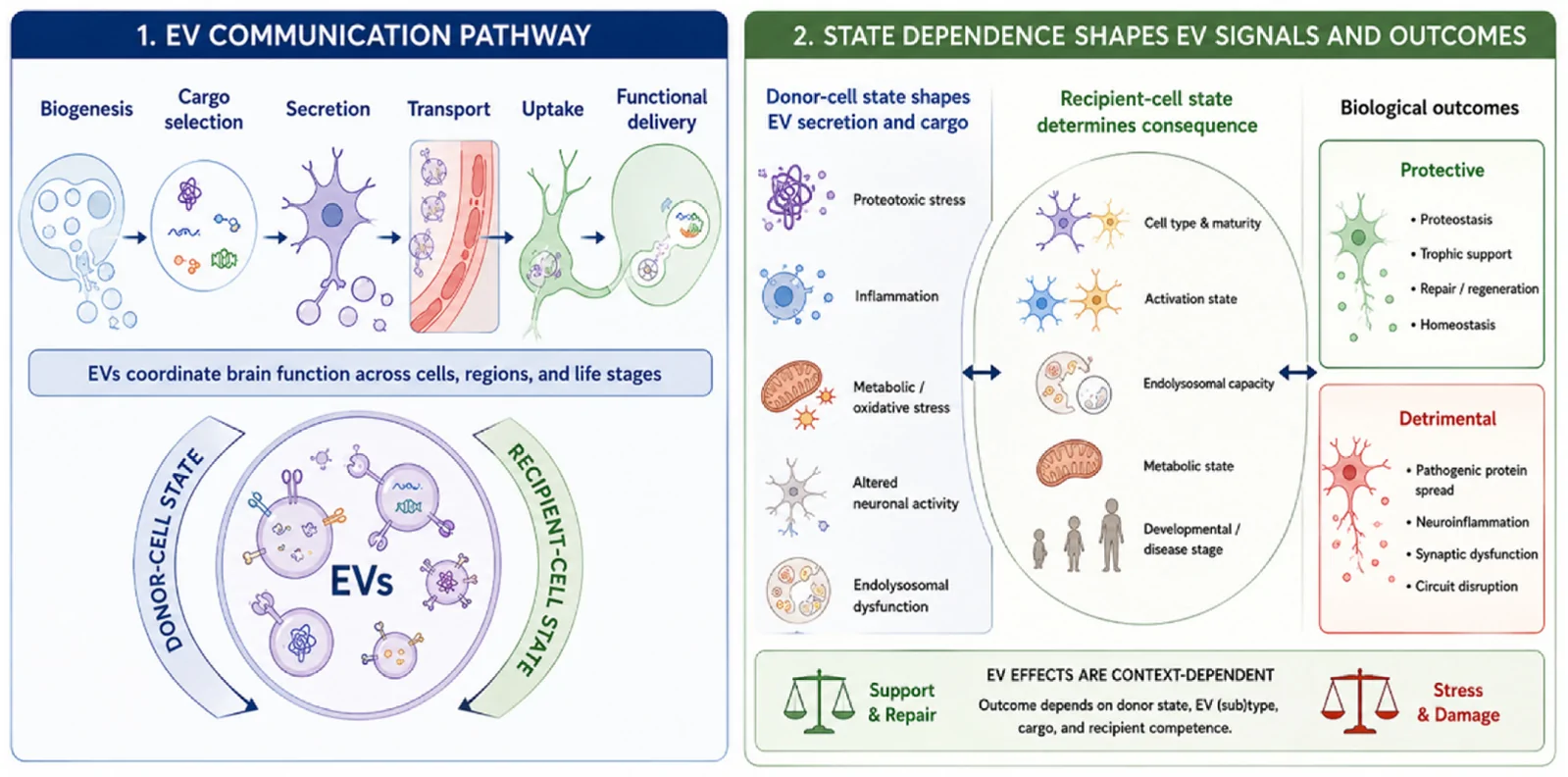
EV communication between different cell types in CNS and the dependency of EV signaling outcomes on donor and recipient cell states.

The evidence reviewed here further indicates that EV communication is shaped by the physiological and pathological states of both donor and recipient cells. In neurodegenerative diseases, proteotoxic stress, inflammation, metabolic and oxidative stress, altered neuronal activity, and endolysosomal dysfunction can modify EV secretion, cargo, and biological activity in a region-, cell-, and EV subtype-dependent manner. In neurodevelopmental disorders, genetic variations can alter EV communication while neural circuits are still developing. Thus, EV abnormalities should not be viewed as fixed signatures of a diagnosis: donor-cell state shapes the EV signal, whereas recipient-cell state helps determine their biological consequences. Both may change with developmental stage, disease progression, anatomical location, and cellular context (Figure 4).

This state dependence may also explain the apparently opposing effects of EVs in neurological diseases. EV secretion can support donor-cell proteostasis by exporting excess or aggregation-prone proteins while simultaneously exposing recipient cells to potentially pathogenic cargo. Conversely, reduced secretion may limit extracellular propagation but increase intracellular protein accumulation and stress. Therefore, whether EV communication is protective or detrimental depends on donor-cell state, cargo, EV subtype, and recipient-cell competence.

Similar considerations apply to EV-associated biomarkers. Cross-sectional differences can identify associations with a disease state but do not necessarily reflect disease progression. Longitudinal biomarker studies require within-person changes that correspond to clinical or biological changes. EV measurements may therefore be most informative when integrated with clinical, imaging, and molecular measures that define the relevant disease or developmental state. Current evidence supports the potential of EVs as components of multimodal biomarker strategies but remains insufficient to establish their general utility as independent measures of disease stage or progression.

EV-based therapeutics must likewise be evaluated across the complete communication pathway. Tissue distribution or cellular uptake alone does not establish functional delivery; therapeutic EVs must reach the appropriate cells, release functional cargo, engage intracellular targets, and produce the intended response. Therapeutic efficacy may therefore depend on recipient-cell competence and disease stage as well as EV source, cargo, dose, and administration route. The same EV preparation may not remain biologically equivalent as neuronal loss, inflammation, or endolysosomal dysfunction progresses.

Future studies should therefore define EV biology across developmental and disease states, use longitudinal sampling for progression claims, and resolve cellular origin and EV subtype whenever possible. Mechanistic studies should distinguish EV production from secretion, uptake from functional cargo delivery, and donor-cell benefit from recipient-cell effects, while moving beyond cargo association to establish causal roles for specific EV components. Therapeutic development should establish cargo causality, intracellular target engagement, stage-dependent efficacy, safety, and relevance to human biology, together with rigorous EV characterization, reproducible manufacturing, biodistribution, and mechanism-linked potency assays [11,81].

The central challenge is no longer simply to detect EVs or define their contents, but to determine when an EV signal is biologically meaningful, how that meaning changes with donor and recipient states, and under what conditions an EV intervention remains effective. Viewing EVs as components of a dynamic, state-dependent communication system provides a testable framework for understanding their roles in CNS physiology and disease and for developing more rigorous EV-associated biomarkers and therapeutics.

## Figures and Tables

**Table 1:** EV-associated biomarker in major neurodegenerative diseases and their interpretive limitations.

Disease	Sample Source/EV Preparation	Candidate Biomarker or EV Feature	Main Finding	Study Context	Potential Relevance and Limitations	Reference
AD	EV preparations reported as neuronal-enriched	EV-associated miRNAs	Altered miRNA profiles were identified in preclinical AD.	Cross-sectional; preclinical disease	Potential early disease-state markers. Neuronal origin requires validation; cross-sectional differences do not establish prediction of progression.	[92]
PD	Salivary EVs	Oligomeric α-synuclein	EV-associated oligomeric α-synuclein and clinical measures remained stable over one year.	Longitudinal	Provides evidence on temporal stability. The study did not establish sensitivity to clinical progression over this interval.	[95]
PD	Plasma EVs	EV cargo measures	Within-person cargo changes were more closely associated with changes in cognition and daily function than baseline levels alone.	Longitudinal; repeated measures	Supports further evaluation for disease monitoring. Associations require independent validation and do not establish predictive performance.	[96]
HD	Brain tissue EV fractions	EV abundance and composition across vesicle subtypes	EV changes differed by neuropathological grade, brain region, and vesicle subtype.	Tissue-based; comparisons across pathological grades	Demonstrates anatomical and stage-related heterogeneity. Tissue findings do not establish an accessible circulating biomarker or within-person progression trajectory.	[22]
ALS	Serum EV preparations	Proteomic and metabolomic profiles	Altered multi-omic profiles were identified in early ALS.	Cross-sectional; early disease	Potential disease-state signatures. Specificity, cellular origin, and longitudinal performance require validation.	[99]
ALS	EV preparations analyzed for TDP-43-related peptides	Cryptic-exon-derived peptides associated with TDP-43 dysfunction	EV-associated peptides reflected a defined disease-related molecular defect.	Molecular biomarker investigation	Potential indicator of TDP-43 dysfunction. Clinical sensitivity, specificity, and relationships with disease stage require further evaluation.	[100]

**Table 3:** Selected clinical trials investigating EV-based therapies for neurological diseases.

Disease	Trial	EV Product and Administration	Evidence to Highlight
Alzheimer’s disease	NCT04388982	Allogeneic adipose MSC-derived exosomes; intranasal administration	Published exploratory study reported no adverse events and preliminary cognitive signals. These findings do not establish efficacy.
Parkinson’s disease	NCT05152394	Allogeneic umbilical cord MSC-derived exosomes; intranasal administration	Registered study evaluating safety and efficacy; no published clinical outcomes were found.
Stroke	NCT05158101	Allogeneic umbilical cord MSC-derived exosomes; intranasal administration	Registered therapeutic study; no published clinical outcomes were found.
Acute ischemic stroke	NCT06138210	Human induced pluripotent stem cell-derived exosomes, GD-iExo-003; intravenous administration	Registered randomized, placebo-controlled, dose-escalation study assessing safety and preliminary efficacy.

MSC, mesenchymal stromal cell. “Exosomes” follows the terminology used by the investigators.

## Data Availability

No new data were created or analyzed in this study. Data sharing is not applicable to this article.

## Abbreviations

The following abbreviations are used in this manuscript:

AS	Angelman syndrome
BBB	Blood–brain barrier
CNS	central nervous system
EV	Extracellular vesicles
ILV	intraluminal vesicle
MVB	multivesicular body
ECM	extracellular matrix m
CSF	cerebrospinal fluid
AD	Alzheimer’s disease
HD	Huntington’s disease
PD	Parkinson’s disease
ALS	Amyotrophic lateral sclerosis
ASD	Autism spectrum disorder
